# A Case of Severe Erythroderma in a Patient with Pustular Psoriasis

**DOI:** 10.5811/cpcem.24854

**Published:** 2024-11-05

**Authors:** Joshua Berko, Christine Raps, Quinlan Cacic, Robert Stephen, Megan Fix, Allison M. Beaulieu

**Affiliations:** University of Utah, Department of Emergency Medicine, Salt Lake City, Utah

**Keywords:** dermatology, pustular psoriasis, erythroderma

## Abstract

**Case Presentation:**

A female patient with a known history of pustular psoriasis presented with sub-acute development of diffuse erythema and scaling of the skin with areas of exfoliation consistent with erythroderma. She was ill appearing and required admission and aggressive treatment with steroid-impregnated wet dressings, topical emollients, analgesics, and systemic immunosuppressants.

**Discussion:**

Erythroderma is a dermatologic emergency characterized by diffuse erythema and scaling spanning greater than 90% of skin surfaces and is associated with a mortality rate as high as 64%. It is initially a clinical diagnosis and needs to be recognized and aggressively treated expeditiously to improve chances of a good outcome.

## CASE PRESENTATION

A 45-year-old female with a history of pustular psoriasis, type II diabetes, thyroid cancer, and endometrial cancer presented to the emergency department (ED) with a diffuse rash. Five weeks prior to presentation she was treated with intramuscular dexamethasone and nirmatrelvir/ritonavir for COVID-19. In the time since her medication administration, she developed a pruritic rash on her abdomen that gradually spread outward toward her extremities. The week prior to presentation, she developed pustules and sloughing of her skin with associated blurred vision, sore throat, chills, and vaginal pain.

**Image 1:**
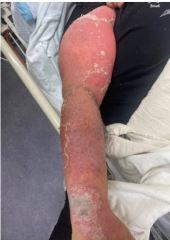
Sloughing, scaling, and erythrodermic skin breakdown of the right arm in the setting of pustular psoriasis.

**Image 2:**
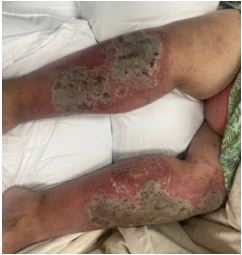
Diffuse scaling overlying erythrodermic reaction of the bilateral lower extremities.

**Image 3:**
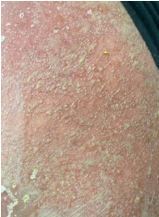
Pustular psoriatic lesions on an erythematous base on the skin of the back.

On physical examination, the patient had a diffuse, erythematous, pustular rash with sloughing (Images 1–3). Nikolsky sign was negative; however, there was evidence of mucosal erythema and sloughing of the genital region, conjunctivae, and oropharynx. Ophthalmology was consulted given concern for toxic epidermal necrolysis and found moderate ocular involvement. They recommended artificial tears and erythromycin ointment. Dermatology was also consulted and performed a biopsy, which later confirmed erythroderma secondary to general pustular psoriasis triggered in the setting of dexamethasone use and COVID-19. The patient was subsequently admitted to the hospital for further treatment, which included infliximab, triamcinolone wet wraps, secukinumab, cephalexin, and gabapentin.

## DISCUSSION

This case highlights an example of pustular psoriasis presenting to the ED with secondary severe erythroderma. Pustular psoriasis is a rare, immune-mediated chronic disease that presents in episodic flares.^1^ Erythroderma is a dermatologic emergency that is characterized by diffuse erythema and scaling involving greater than 90% of skin surfaces and is associated with a mortality rate as high as 64%.^2^ The most common causes are exacerbation of pre-existing dermatosis and drug reactions, but it can also be seen with infections and other systemic diseases.^2^–^4^ Symptoms include diffuse skin involvement, notably pruritic, scaling, crusting, erythematous patches with progressive exfoliation and may be associated with systemic symptoms such as shivering, hypothermia, fever, malaise, peripheral edema, and tachycardia.^4^

**CPC-EM Capsule**

What do we already know about this clinical entity?

*Erythroderma presents as diffuse erythema, scaling, and progressive exfoliation of over 90% of skin surface with fever, malaise, tachycardia, and peripheral edema.*

What is the major impact of the image(s)?

*Diffuse erythema with scaling and sloughing of skin in the setting of a known history dermatoses such as pustular psoriasis aids in the diagnosis.*

How might this improve emergency medicine practice?

*Awareness of this rare disease will aid in the early, correct diagnosis and treatment, which may decrease mortality and morbidity.*

Erythroderma can have a variable onset and is initially diagnosed clinically^4^; therefore, history should focus on identifying triggers or genetic factors by asking about prior skin conditions, medications, and family history. Physical exam should include a complete skin and mucosal exam to identify extent of involvement and evaluate for secondary infection. Laboratory testing should include a complete blood count, which commonly reveals leukocytosis. A comprehensive metabolic panel is useful in assessing electrolyte status, glucose and albumin levels, lactate dehydrogenase, and kidney and liver function.^4^ If systemic or superimposed infection is suspected, bacterial and fungal cultures, and a viral polymerase chain reaction for herpes simplex virus or varicella zoster virus may be warranted.

Initial treatment for all causes of erythroderma include hemodynamic management, fluid and electrolyte replacement, wound care, temperature management, nutritional support, treatment of superimposed infections, and symptomatic management with wet dressings, emollients, oral antihistamines (eg, hydroxyzine hydrochloride), and low to medium dose systemic prednisone (0.5–1 milligram per kilogram per day with taper) or topical steroids (clobetasol 0.05% or triamcinolone twice daily for 2–4 weeks).^2^–^4^ Emergency physicians need to be able to recognize erythroderma early to rapidly provide lifesaving, stabilizing treatment and admission.
